# Enhanced efficacy of lung cancer treatment with radiotherapy and immune checkpoint inhibitors without increased pneumonia risk: a systematic review and meta-analysis of randomized controlled trials

**DOI:** 10.3389/fimmu.2025.1685963

**Published:** 2025-12-08

**Authors:** Wenjing Wang, Lisha Ye, Yun Chen, Huihui Li, Weimin Mao, Xiaoling Xu

**Affiliations:** 1 Zhejiang Cancer Hospital, Hangzhou Institute of Medicine (HIM), Chinese Academy of Sciences, Hangzhou, Zhejiang, China; 2 Postgraduate Training Base Alliance, Wenzhou Medical University, Wenzhou, Zhejiang, China; 3 Department of Radiation Oncology, Shanghai Pulmonary Hospital, Tongji University School of Medicine, Shanghai, China; 4 Department of Gastroenterology, Sanmen People’s Hospital, Taizhou, Zhejiang, China; 5 Zhejiang Provincial Key Laboratory of Diagnosis and Treatment of Thoracic Cancer, Hangzhou, Zhejiang, China

**Keywords:** lung cancer, radiotherapy, immune checkpoint inhibitors, pneumonia, survival

## Abstract

**Objective:**

Combined modality treatment with chemotherapy, radiotherapy, and immunotherapy is a crucial therapeutic approach for lung cancer. However, controversies still exist regarding radiation doses, treatment regimens, and the risk of pneumonitis. This study aimed to conduct a comprehensive meta-analysis and in-depth subgroup analyses based on randomized controlled trials (RCTs) involving lung cancer patients undergoing radiotherapy to assess whether its combination with immunotherapy is effective and safe.

**Methods:**

We systematically searched PubMed, Cochrane Central, Embase, and major conferences for randomized trials evaluating immune checkpoint inhibitors (ICIs) plus radiotherapy in lung cancer. The outcomes included progression-free survival (PFS), overall survival (OS), and the incidence of adverse reactions, particularly focusing on pneumonitis/pneumonia. Subgroup analyses were performed based on radiotherapy modalities, the timing of ICIs treatment, tumor stage, pathological type, and types of ICIs.

**Results:**

Fifteen trials were included in this analysis. The addition of ICIs to radiotherapy or chemoradiotherapy significantly improved PFS (HR = 0.76, 95% CI 0.70–0.83) and OS (HR = 0.83, 95% CI 0.75–0.92). In subgroup analyses, stereotactic body radiotherapy (SBRT) (HR = 0.38, 95% CI 0.19-0.75) and hypo-fractionated radiotherapy (Hypo-RT) (HR = 0.49, 95% CI 0.31-0.79) were associated with improved PFS. Consolidation ICIs treatment improved OS (HR = 0.68, 95% CI 0.59-0.79), while concurrent ICIs had no significant effect (HR = 1.06, 95% CI 0.87-1.28). In terms of tumor stage, Stage I NSCLC patients (HR = 0.38, 95% CI 0.19-0.75) showed significant PFS improvement with ICIs. Both PD-1 (HR = 0.39, 95% CI 0.22-0.69) and PD-L1 (HR = 0.75, 95% CI 0.64-0.87) inhibitors were linked to improved PFS in irradiated lung cancer patients, and PD-L1 also enhanced OS (HR = 0.82, 95% CI 0.68-0.99). The addition of ICIs increased the risk of any-grade pneumonitis/pneumonia (RR = 1.27, 95% CI 1.12-1.44) but did not elevate the risk of severe (grade ≥3) events (RR = 1.12, 95% CI 0.78-1.60). Notably, among patients treated with SBRT, no significant increase was observed in the incidence of pneumonitis of any grade.

**Conclusions:**

PD-1/PD-L1 inhibitors combined with radiotherapy especially SBRT can enhance survival outcomes in lung cancer without increasing the risk of severe pneumonitis/pneumonia, supporting their clinically manageable safety profile.

**Systematic Review Registration:**

https://www.crd.york.ac.uk/PROSPERO/home, identifier CRD420251140111.

## 1 Introduction

Lung cancer remains one of the most prevalent cancers worldwide and is a leading cause of cancer-related mortality, with nearly two million new cases and 1.76 million deaths annually. Non-small cell lung cancer (NSCLC) constitutes approximately 85% of these cases (1), with about half being detected at an early stage and nearly one-third diagnosed with locally advanced disease. Radiotherapy serves as a primary treatment for early-stage patients, particularly when surgical interventions are not feasible. Advanced techniques such as stereotactic body radiation therapy (SBRT) are employed to minimize damage to surrounding healthy tissues. For patients with advanced-stage cancer, radiotherapy provides palliative relief from symptoms, including pain and breathing difficulties caused by tumor compression. Additionally, post-operative radiotherapy plays a crucial role in eradicating residual microscopic cancer cells, thereby reducing the risk of recurrence.

Recent advances in combining radiotherapy with immune checkpoint inhibitors (ICIs), particularly PD-1/PD-L1 inhibitors, have shown promising therapeutic outcomes. The PACIFIC trial, a landmark phase III trial, investigated the use of ICIs as consolidation therapy in patients with locally advanced NSCLC (LA-NSCLC) who exhibited no disease progression following concurrent chemoradiotherapy(cCRT) with platinum-based chemotherapy (2). Results from this trial demonstrated that durvalumab consolidation therapy significantly improved progression-free survival (PFS) and overall survival (OS) compared to placebo. Durvalumab has thus been approved for treating stage III NSCLC patients who are inoperable and have not progressed after platinum-based chemoradiotherapy. However, subsequent studies, such as PACIFIC-2, have yielded less favorable outcomes, leaving the efficacy of combining radiotherapy with immunotherapy in lung cancer treatment subject to ongoing debate. The latest PACIFIC-5 trial results have reaffirmed the significant benefits of the PACIFIC regimen, providing crucial evidence-based support for the use of durvalumab as consolidation therapy after chemoradiotherapy in patients with unresectable stage III NSCLC.

The combination of differing therapeutic modalities, however, may incur costs in terms of increased toxicity and safety concerns, particularly concerning pulmonary tissue damage. Radiation pneumonitis, an early manifestation of radiation-induced lung injury, affects approximately 10%-30% of patients undergoing thoracic radiotherapy (3). Moreover, immunotherapy alone can lead to severe immune-related pneumonitis, with incidence rates ranging from 3.1% to 4.1% in NSCLC patients (4). The potential escalation in pneumonia incidence, and particularly severe cases, when radiotherapy is paired with immunotherapy remains a pressing concern among clinician. Hence, determining whether combination therapy truly heightens the risk of radiation pneumonitis or pneumonia is a critical clinical issue warranting thorough investigation.

Above all, there is ongoing debate regarding the efficacy and safety of combining ICIs with radiotherapy in the treatment of lung cancer. The incidence of pneumonitis persists as a major clinical concern, alongside the need for detailed subgroup analyses to identify the specific populations likely to benefit from this approach. While some studies have synthesized the efficacy and adverse reactions associated with the combination of radiotherapy and immunotherapy (5), most are retrospective, limiting the solidity of the evidence. In light of several recent major studies, we conducted a systematic review and meta-analysis of randomized controlled trials (RCTs) involving lung cancer patients undergoing radiotherapy to assess whether its combination with immunotherapy offers survival benefits and increases the incidence of radiation-induced pneumonia in patients treated with ICIs in conjunction with radiotherapy.

## 2 Methods

This systematic review and meta-analysis investigated immunotherapy strategies for lung cancer patients undergoing radiotherapy, following the PRISMA guidelines (**Supplementary Table S1**).

### 2.1 Eligibility

Studies fulfilling the following criteria were considered eligible for inclusion: all published and unpublished RCTs; enrollment of histologically or cytologically confirmed lung cancer; RCT clinical trials comparing immunotherapy inclusion based on radiotherapy treatment strategies, with clinical outcomes including at least PFS, OS, any level of radiation pneumonitis/pneumonia or severe (Grade 3 or higher) adverse events. Studies that do not meet the inclusion criteria are excluded. Additional exclusion criteria include retrospective studies, single-arm studies, and other study designs, as well as trials involving subjects who have previously received ICIs therapy.

### 2.2 Search strategy

We conducted searches across clinical trial databases, including PubMed, Cochrane Central, and Embase, from inception through October 2025. Search terms comprised immunotherapy, PD-1, PD-L1, radiotherapy, and lung cancer, aiming to identify RCTs that compare the efficacy of combining immunotherapy with radiotherapy versus radiotherapy alone in treating lung cancer. We also reviewed abstracts and reports from major conferences such as ASCO, ESMO, ELCC and WCLC up to October 2025 and examined reference lists from recent related reviews and meta-analyses to ensure comprehensiveness.

### 2.3 Data extraction and analysis

Extracted data encompassed characteristics of the studies (study ID, phase status, publication year, and sample size), demographic details (gender, age, race, smoking status), treatment modalities, and outcomes (hazard ratios for PFS and OS with their 95% confidence intervals (CIs), along with any grade of radiation pneumonitis/pneumonia and other adverse events). Where possible, we prioritized data assessed by a blinded, independent review committee, adhering to the intention-to-treat principle, and prioritized the most recent data from multiple reports of the same trial, provided they had different follow-up times. In instances where multiple publications from the same study existed, only the most recent and complete reports, or those supporting Food and Drug Administration (FDA) approvals, were included. For any data gaps encountered, **Supplementary Materials** were reviewed, and corresponding authors contacted as needed.

Extract the following data from each study: the first author’s name, year of publication, number of patients, gender, pathological type, stage, type of radiotherapy, type of ICIs, treatment sequence, and incidence of radiation pneumonitis/pneumonia. In instances where the number of radiation pneumonitis cases is not reported, the number of pneumonia cases was used as a substitute. Survival outcomes were also collected.

All statistical analyses were conducted using R software (version 4.3.0), utilizing the Meta package for meta-analysis. The primary outcomes analyzed were PFS, OS, and any level or severity of radiation pneumonitis/pneumonia. We considered a P-value of less than 0.05 as indicative of statistical significance. The data analysis period spanned from October 5, 2024, to October 1, 2025.

## 3 Result

**Figure 1:**
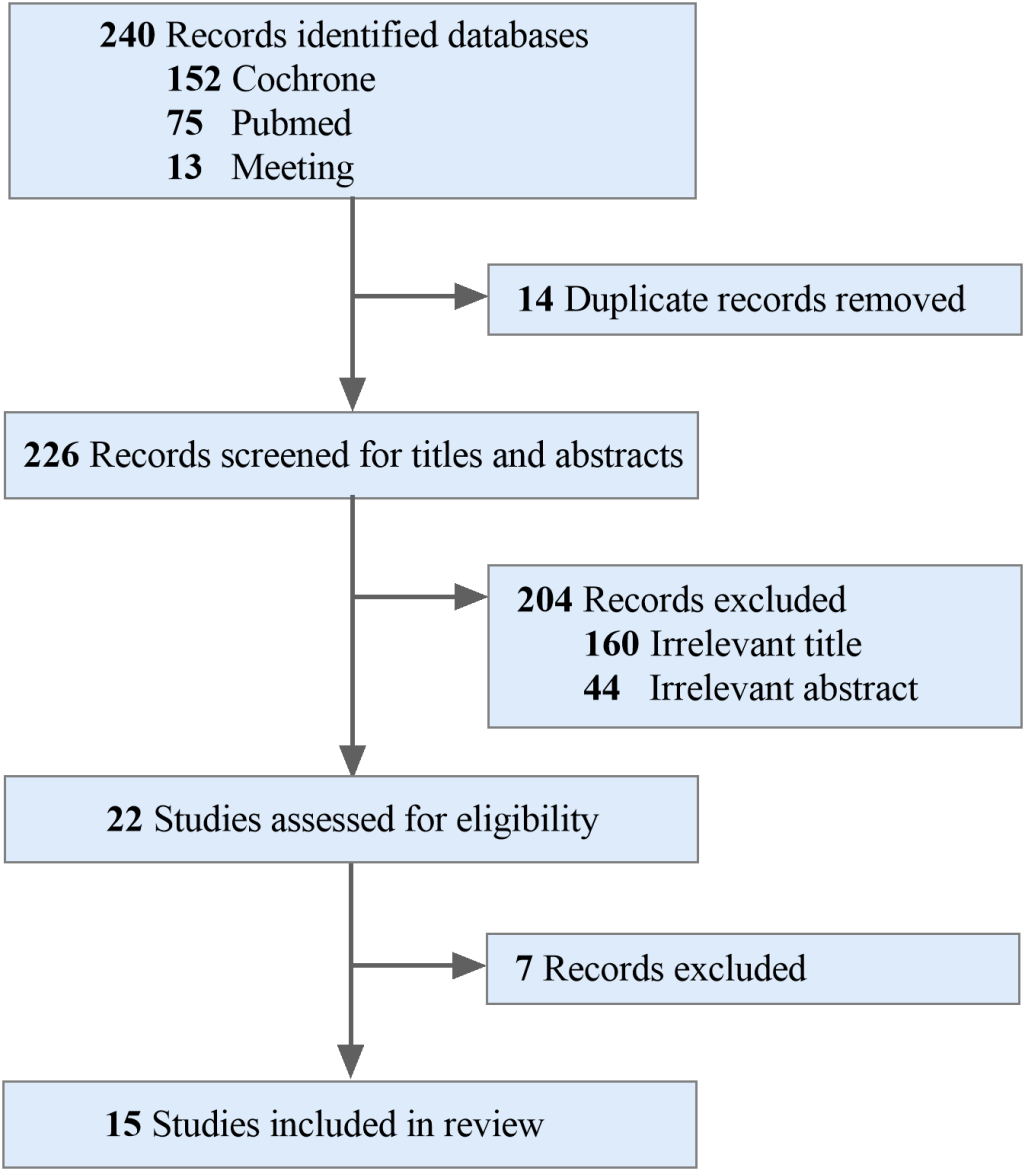
PRISMA flow diagram of study selection for meta-analysis.

In the initial search, 240 researches were identified. Through a comprehensive analysis of titles and abstracts, we recognized 22 studies exploring the effects of combined radiotherapy and ICIs on survival outcomes and patient prognosis. 7 studies were excluded for not meeting the inclusion criteria. Ultimately, 15 studies that met the established criteria were selected for inclusion in the meta-analysis, encompassing a total of 3,769 patients (**Figure 1**).

The characteristics of each study are summarized in **Table 1**. Of these, 13 studies provided data on PFS, ten on OS, and 14 studies on radiation pneumonitis or pneumonia. Regarding radiotherapy modalities, the majority of studies concentrated on conventional radiotherapy. Most research centered on the sequence of ICIs administration, specifically consolidation and concurrent therapy. Concerning cancer staging, the studies primarily addressed stage III lung cancer and SCLC. In terms of ICIs, the focus was predominantly on PD-L1 inhibitors.

**Table 1:** The characteristics of the studies included in the systematic review.

Number	Clinical trial	Phase	Clinical Stage	Histopathological type	RT	ICIs types	ICIs sequecing	Sample size	Median age	Sex	Race	ECOG PS	Smoking history	Histology	PD-L1 expression	Survival outcomes
1	Chang, J. Y (26). (2023)	II	I	NSCLC	SBRT	Control group (/)	/	75	72 (66-78)	F-41 (55%) M-34(45%)	White-64(85%) Any other race-11 (15%)	0~1-68 (91%) 2-7 (9%)	Never-7 (9%) Current or previous-68 (91%)	NSC-61 (81%) SCC-14 (19%)	<1%-34 (45%) ≥1%-16 (21%) Unknown-25 (33%)	Event-free survival rate, AEs
						Trial group (Nivo) (PD-1)	Concurrent	66	72 (66-75)	F-46 (70%) M-20(30%)	White-62 (94%) Other-4 (6%)	0~1-62 (94%) 2-4 (6%)	Never-7 (11%) Current or previous-59 (89%)	NSC-55 (83%) SCC-11 (17%)	<1%-27 (41%) ≥1%-15 (23%) Unknown-24 (36%)	
2	ASTEROID trial (2021)	II	I	NSCLC	SBRT	Control group (/)	/	25	76(58-89)	F-15 (60%) M-10(40%)	/	0-8 1-11 2-6	Never-1 Former-19 On-going-5	AC-16 SCC-8 NOS-1	/	PFS
						Trial group (Durva) (PD-L1)	Adjust	22	77(69-86)	F-16 (72.7%) M-6(27.3%)	/	0-5 1-13 2-4	Never-0 Former-17 On-going-5	AC-15 SCC-5 NOS-2	/	
3	PACIFIC trial (27, 28) (2022)	III	III	NSCLC	Con-RT	Control group (Placebo)	/	237	64(23-90)	F-71 (30.0%) M-166(70.0%)	White-157 (66.2%) Black-2 (0.8%) Asian-72 (30.4%)	0-114 (48.1%) 1-122 (51.5%)	Never-21(8.9%) Former-178(75.1%) Current-38(16.0%)	NSC-135 (57.0%) SCC-102 (43.0%)	≥25%-44 <25%-105 Unknown-88	PFS, OS
						Trial group (Durva) (PD-L1)	Consolidation	476	64(31-84)	F-142 (29.8%) M-334(70.2%)	White-337 (70.8%) Black-12 (2.5%) Asian-120 (25.2%)	0-234 (49.2%) 1-240 (50.4%)	Never-43(9.0%) Former-354(74.4%) Current-79(16.6%)	NSC-252 (52.9%) SCC-224 (47.1%)	≥25%-115 <25%-187 Unknown-174	
4	PACIFIC-2 trial (2023)	III	III	NSCLC	Con-RT	Control group (Placebo)	/	109	63.0 (38-84)	F-29 (26.6%) M-80(73.4%)	White-62 (56.9%) Black or African American-0 Asian-39 (35.8%) American Indian or Alaska Native-7 (6.4%) Other-1(0.9%)	0-53 (48.6%) 1-56 (51.4%)	/	NSC-135 (57.0%) SCC-102 (43.0%)	≥25%-44 <25%-105 Unknown-88	PFS, therapeutic response
						Trial group (Durva) (PD-L1)	Concurrent	219	63.0 (36-84)	F-53 (24.2%) M-166(75.8%)	White-141 (64.4%) Black or African American-2 (0.9%) Asian-65 (29.7%) American Indian or Alaska Native-7 (3.2%) Other-4(1.8%)	0-98 (44.7%) 1-121 (55.3%)	/	NSC-98 (44.7%) SCC-121 (55.3%)	<1% (negative)-86 (39.3%) ≥1% (positive)-113 (51.6%) Unkown-20 (9.1%)	
5	PACIFIC-5 trial (2024)	III	III	NSCLC	Con-RT	Control group (Placebo)	/	129	63.0 (33-79)	F-15 (11.6%) M-114(88.4%)	Asian-94 (72.9%) Non-Asian-35 (27.1%)	0-58 (45.0%) 1-71 (55.0%)	Never-15 (11.6%) Current or Former-114 (88.4%)	SCC-84 (65.1%) AC-40 (31.0%) LCC-0 Other-0	<1%-51 (39.5%) ≥1%-78 (60.5%)	PFS
						Trial group (Durva) (PD-L1)	Consolidation/concurrent	252	63.0 (39-80)	F-20 (7.9%) M-232(92.1%)	Asian-180 (71.4%) Non-Asian-72 (28.6%)	0-80 (31.7%) 1-172 (68.3%)	Never-38 (15.1%) Current or Former-214 (84.9%)	SCC-176 (69.8%) AC-64 (25.4%) LCC-3 (1.2%) Other-9 (3.6%)	<1%-101 (40.1%) ≥1%-151 (59.9%)	
6	DETERRED trial (17, 29) (2022)	II	III	NSCLC	Con-RT	Control group (/)	/	10	66(52-71)	F-1 (10.0%) M-9(90.0%)	Caucasian-8 (80%) African Americans-2 (20%)	/	Never-0 (0%) Ever-9 (90%) Current-1 (10%)	AC-2 (20%) SCC-7 (70%) Other/not specified-1 (10%)	≥1%-4 (44%) ≥50%-2 (22%) Unknown-1	AEs
						Trial group (Atez) (PD-L1)	Concurrent	30	68(50-83)	F-12 (40.0%) M-18(60.0%)	Caucasian-28 (93%) African Americans-2 (7%)	/	Never-9 (30%) Ever-14 (47%) Current-7 (23%)	AC-20 (67%) SCC-7 (23%) NSCLC-3 (10%)	≥1%-11 (44%) ≥50%-6 (24%) Unknown-5	
7	SPRINT trial (30) (2022)	II	III	NSCLC	PRT	Control group (/)	/	12	69(60–82)	F-5 (42.0%) M-7(58.0%)	/	0-2(17%) 1-10(83%)	/	AC-4(33%) SCC-6(50%) Other -2(17%)	18% (4% to 34%)	One-year PFS
						Trial group (Pembro) (PD-1)	Induction	25	70(53-86)	F-12 (48.0%) M-18(60.0%)	/	0-8(32%) 1-17(68%)	/	AC-11 (44%) SCC-11 (44%) Other -3(12%)	75% (70% to 80%)	
8	ALLSTAR trial (31) (2024)	III	III	NSCLC	Con-RT	Control group (/)	/	58	68(36-91)	F-22 (38.0%) M-36(62.0%)	/	0~1-53 (91%) 2~3-5 (9%)	Never-3 (5%) Ever-33 (57%) Current-22 (38%)	AC-26 (45%) SCC-26 (45%) NOS-6 (10%)	<1%-26 (45%) >1%-25 (43%) Unknown-7 (12%)	PFS, local control rate, AEs
						Trial group (Durva/Pembro/Nivo/Atez) (unlimited)	Consolidation	130	67(41-84)	F-52 (40.0%) M-78(60.0%)	/	0~1-123 (95%) 2~3-7 (5%)	Never-10 (8%) Ever-75 (58%) Current-45 (35%)	AC-72 (55%) SCC-52 (40%) NOS-5 (4%)	<1%-17 (13%) >1%-105 (81%) Unknown-8 (6%)	
9	GEMSTONE-301 trial (32) (2022)	III	III	NSCLC	Con-RT	Control group (Placebo)	/	126	60(55-65)	F-11 (9.0%) M-115(91.0%)	/	0-38 (30%) 1-88 (70%)	Former or Current-110 (87%) Never-16 (13%)	NSC-40 (32%) SCC-86 (68%) Missing data-0	/	PFS
						Trial group (Sugemalimab) (PD-L1)	Consolidation	255	61(56-65)	F-19 (7.0%) M-236(93.0%)	/	0-78 (31%) 1-177 (69%)	Former or Current-213 (84%) Never-42 (16%)	NSC-76 (30%) SCC-177 (69%) Missing data-2 (1%)	/	
10	GASTO-1091 trial (35) (2024)	II	III	NSCLC	Hypo-RT	Control group (/)	/	86	62(30-75)	F-16 (18.6%) M-70(81.4%)	/	0-55(64.0%) 1-31(36.0%)	Non-smoker-29(33.7%) Smoker-57(66.3%)	SCC-45(52.3%) NSC-37(43.0%) NOS-4(4.7%)	<1%-20(23.3%) ≥1%-30(34.9%) Missing-36(41.9%)	PFS
						Trial group (Nivo) (PD-L1)	Consolidation	86	57(39-73)	F-15 (17.4%) M-71(82.6%)	/	0-57(66.3%) 1-29(33.7%)	Non-smoker-28(32.6%) Smoker-58(67.4%)	SCC-38(44.2%) NSC-43 (50.0%) NOS-5(5.8%)	<1%-23(26.7%) ≥1%-38 (44.2%) Missing-25 (29.1%)	
11	J. A. Moran (33) (2022)	Prospective	IV	NSCLC	unlimited	Control group (/)	/	41	66(45-78)	F-18 (43.9%) M-23(56.1%)	Caucasion-32(78.0%) Blank-5(12.2%) Others-4(9.8%)	0-18(43.9%) ≥1-21(51.2%) Unknown-2(4.9%)	Heavy-9(21.9%) Light-23(56.2%) Never-9 (21.9%) Unknown-0	AC-27 (65.9%) SCC-9 (21.9%) NSCLC-5(12.2%)	Available tumor PD-L1-15(36.6%) <50%-15(100.0%) ≥50%-0	PFS
						Trial group (Pembro/Nivo/Atez) (unlimited)	Ever	41	63(45-81)	F-17 (41.5%) M-24(58.5%)	Caucasion-33(80.5%) Blank-5(12.2%) Others-3(7.3%)	0-17(41.5%) ≥1-21(51.2%) Unknown-3(7.3%)	Heavy-14(34.1%) Light-19(46.3%) Never-5 (12.2%) Unknown-3(7.4%)	AC-21 (51.2%) SCC-8 (19.5%) NSCLC-12(29.3%)	Available tumor PD-L1-19(45.2%) <50%-10(52.6%) ≥50%-9(47.4%)	
12	ADRIATIC trial (34) (2024)	III	Limited	SCLC	Con-RT	Control group (Placebo)	/	266	62(28-79)	F-78 (29.3%) M-188(70.7%)	White-51.5% Asian-45.5% Other-3.0%	0-47.4% 1-52.6%	Never-9.8% Former-69.5% Current-20.7%	/	/	OS, PFS
						Trial group (Durva) (PD-L1)	Consolidation	264	62(28-84)	F-86 (32.6%) M-178(67.4%)	White-49.2% Asian-49.6% Other-1.1%	0-50.0% 1-50.0%	Never-8.7% Former-67.4% Current-23.9%	/	/	
13	NRG LU005 trial (18) (2024)	III	Limited	SCLC	Con-RT	Control group (/)	/	254	/	/	/	/	/	/	/	OS
						Trial group (Atez) (PD-L1)	Concurrent	267	/	/	/	/	/	/	/	
14	Xu Y. (36) (2024)	II	Limited	SCLC	Con-RT	Control group (/)	/	19	61(48-72)	F-2 (10.5%) M-17 (89.5%)	/	0-4(21.1%) 1-12(63.2%) Unknown-3(15.8%)	Never-4(21.1%) Former-14(73.7%) Other-1(5.3%)	/	/	1-year PFS rate
						Trial group (Camre) (PD-1)	consolidation	19	64(30-73)	F-2 (10.5%) M-17 (89.5%)	/	0-7(36.8%) 1-12(63.2%) Unknown-0	Never-4(21.1%) Former-15(78.9%)	/	/	
15	Bjom H. Gronberg		Limited	SCLC	Con-RT	Control group (/)	/	85		F-36 (42.4%) M-49 (57.6%)	/	/	/	/	/	PFS, OS
						Trial group (Atez) (PD-L1)	adjust	85		F-43 (50.6%) M-42 (49.4%)	/	/	/	/	/	

AC, Adenocarcinoma. AEs, adverse events. Atez, Atezolizumab. Camre, Camrelizumab. Con-RT, conventional radiotherapy. Durva, Durvalumab. F, female. Hypo-RT, high dose fractionated radiotherapy. IMRT, intensity-modulated radiation therapy. LCC, large cell carcinoma. M, male. N, the number of participants. Nivo, Nivolumab. OS, overall survival. Pembro, Pembrolizumab. PFS, progression free survival. PRT, proton radiation therapy. RT, radiation treatment. SBRT, stereotactic body radiation therapy. SCC, squamous cell carcinoma. SCLC, small cell lung cancer.

### 3.1 The impact of combining radiotherapy with immune checkpoint inhibitors on the survival of lung cancer patients

**Figure 2:**
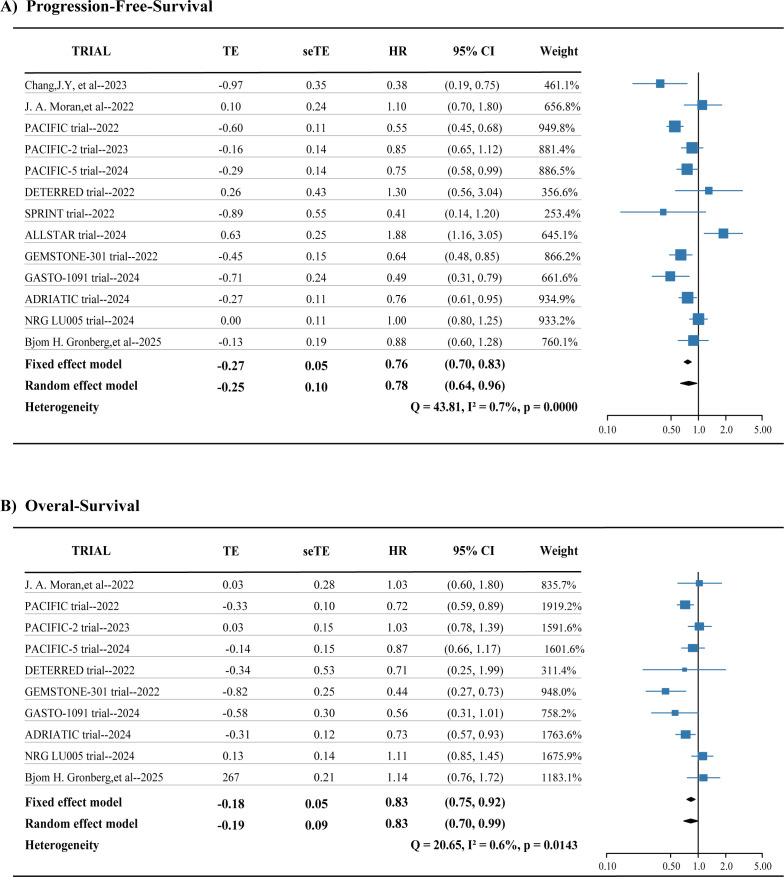
**(A, B)** Pooled hazard ratios (HRs) of progression-free-survival and overall survival across randomized clinical trials. I2 is a statistical value for assessing heterogeneity. CI, confidence interval. HR, hazard ratio.

Our meta-analysis included 13 studies on PFS, which demonstrated that adding ICIs to radiotherapy improves PFS in lung cancer patients (HR = 0.76, 95% CI 0.70-0.83) (**Figure 2A**). Similarly, the analysis of OS encompassed 10 studies and showed an enhancement in OS with the incorporation of ICIs (HR = 0.83, 95% CI 0.75-0.92) (**Figure 2B**).

### 3.2 Subgroup analysis for survival

Patients receiving SBRT (HR = 0.38, 95% CI 0.19-0.75), conventional radiotherapy (Con-RT) (HR = 0.76, 95% CI 0.64-0.89), and high-dose fractionated radiotherapy (Hypo-RT) (HR = 0.49, 95% CI 0.31-0.79) exhibited improvements in PFS. Conversely, proton radiation therapy (PRT) (HR = 0.77, 95% CI 0.25-2.37) showed no significant impact on PFS with the addition of ICIs. As for OS, Con-RT (HR = 0.84, 95% CI 0.69-1.03) and Hypo-RT (HR = 0.56 95% CI 0.31-1.01) had potential benefits in improving patient outcomes, whereas findings indicated that PRT (HR = 0.71, 95% CI 0.25-1.99) did not produce statistically significant effects.

Neither concurrent ICIs treatment (HR = 0.83, 95% CI 0.57-1.21), consolidation ICIs treatment (HR = 0.74, 95% CI 0.48-1.14), nor induction ICIs treatment (HR = 0.41, 95% CI 0.14-1.20) demonstrated improvement in PFS for patients. Conversely, consolidation ICIs treatment (HR = 0.68, 95% CI 0.59-0.79), whereas concurrent ICIs treatment did not show a statistically significant impact on OS (HR = 1.06, 95% CI 0.87-1.28).

Patients with Stage III (HR = 0.76, 95% CI 0.56-1.04), Stage IV NSCLC (HR = 1.10, 95% CI 0.70-1.80), as well as SCLC (HR = 0.87, 95% CI 0.75-1.01, who received radiation therapy showed no statistically significant improvement in PFS upon receiving ICIs treatment. Conversely, in Stage I NSCLC patients (HR = 0.38, 95% CI 0.19-0.75), the addition of immunotherapy significantly improved PFS. Regarding OS, ICIs treatment significantly enhanced OS in patients with Stage III NSCLC (HR = 0.74, 95% CI 0.57-0.94). However, in patients with Stage IV NSCLC (HR = 1.03, 95% CI 0.60-1.80) and SCLC (HR = 0.95, 95% CI 0.71-1.29), no statistically significant effect on OS was observed.

Both PD-1 (HR = 0.39, 95% CI 0.22-0.69) and PD-L1 (HR = 0.75, 95% CI, 0.64-0.87) were associated with improved PFS in lung cancer patients undergoing radiation therapy. For OS, PD-L1 was shown to enhance OS in these patients (HR = 0.82, 95% CI, 0.68-0.99) (**Table 2**).

**Table 2:** Subgroup analyses of progression-free and overall survival according to study characteristics and methods.

Characteristic	Progression-free survival		Overall survival	
	Hazard ratio	95% CI	Hazard ratio	95% CI
Radiotherapy rodalities				
SBRT	0.38	[0.19, 0.75]	/	/
Con-RT	0.76	[0.64, 0.89]	0.84	[0.69, 1.03]
PRT	0.77	[0.25, 2.37]	0.71	[0.25, 1.99]
Hypo-RT	0.49	[0.31, 0.79]	0.56	[0.31, 1.01]
Timing of combination therapy with Immunotherapy				
Concurrent	0.83	[0.57, 1.21]	1.06	[0.87, 1.28]
Consolidation	0.74	[0.48, 1.14]	0.68	[0.59, 0.79]
Induction	0.41	[0.14, 1.20]	/	/
Adjust	0.88	[0.60, 1.28]	1.14	[0.76, 1.72]
Clinical Stage				
I	0.38	[0.19, 0.75]	/	/
III	0.76	[0.56, 1.04]	0.74	[0.57, 0.94]
IV	1.10	[0.70, 1.80]	1.03	[0.60, 1.80]
Limited	0.87	[0.75, 1.01]	0.95	[0.71, 1.29]
Histology				
NSCLC	0.75	[0.56, 1.00]	0.77	[0.61, 0.96]
SCLC	0.87	[0.75, 1.01]	0.95	[0.71, 1.29]
ICI type				
PD-1	0.39	[0.22, 0.69]	/	/
PD-L1	0.75	[0.64, 0.87]	0.82	[0.68, 0.99]
Total	**0.76**	**[0.70, 0.83]**	**0.83**	**[0.75, 0.92]**

SBRT, stereotactic body radiation therapy. Con-RT, conventional radiotherapy. IMRT, intensity-modulated radiation therapy. PRT, proton radiation therapy. Hypo-RT, high dose fractionated radiotherapy. SCLC, small cell lung cancer.

### 3.3 The impact of combining radiation therapy with immunotherapy on the incidence of radiation pneumonitis/pneumonia

**Figure 3:**
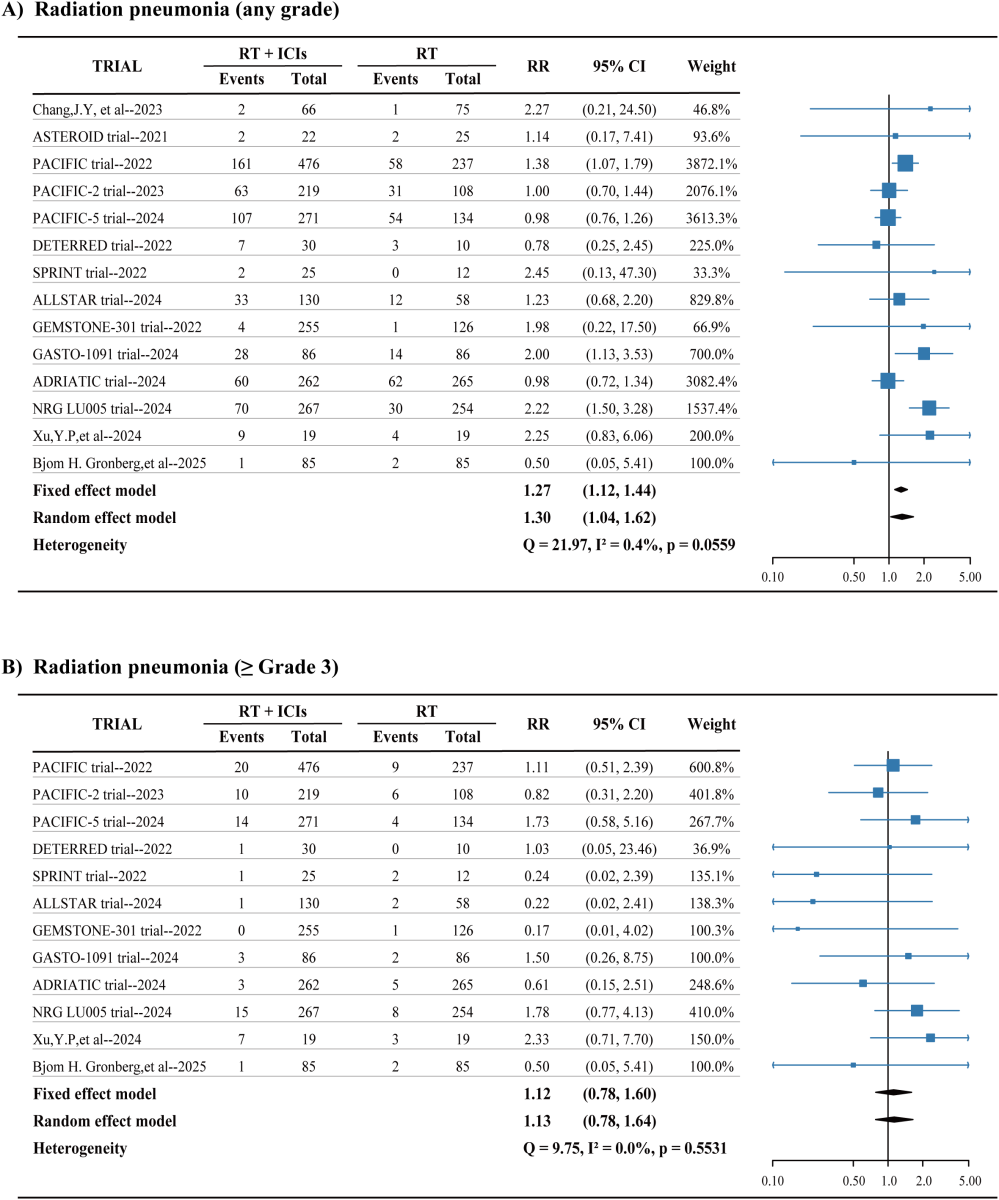
**(A, B)** Pooled risk ratios (RRs) of radiation pneumonia across randomized clinical trials. I2 is a statistical value for assessing heterogeneity. CI, confidence interval. RR, risk ratio.

In the studies reviewed, 14 reported on the incidence of any grade radiation pneumonitis/pneumonia in both the group receiving only radiation therapy and the group treated with radiation combined with ICIs, involving a total of 3,707 patients. The analysis indicated that the addition of ICIs treatment was associated with an increased risk of any grade radiation pneumonitis/pneumonia (RR = 1.27, 95% CI 1.12-1.44) (**Figure 3A**). Meanwhile, 12 studies reported on the incidence of severe radiation pneumonitis/pneumonia (Grade ≥3) across both groups, covering 3,519 patients. These results did not show a statistically significant association between the addition of ICIs and an increased risk of grade 3 or higher radiation pneumonitis/pneumonia (RR=1.12, 95% CI 0.78-1.60) (**Figure 3B**).

### 3.4 Subgroup analysis for the incidence of radiation pneumonitis/pneumonia

The addition of ICIs did not significantly impact the incidence of any grade of radiation pneumonitis/pneumonia for patients treated with SBRT (RR = 1.52, 95% CI 0.35-6.48), Con-RT (RR = 1.26, 95% CI 0.96-1.66) or PRT (RR = 1.00, 95% CI 0.34-2.91). However, Hypo-RT (RR = 2.00, 95% CI 1.13-3.53) were associated with an increased risk of any grade of radiation pneumonitis/pneumonia. Regarding severe radiation pneumonitis/pneumonia (Grade ≥3), no statistically significant effects were observed with Con-RT (RR = 1.21, 95% CI 0.82-1.77), PRT (RR = 0.42, 95% CI 0.07-2.37), or Hypo-RT (RR = 1.50, 95% CI 0.26-8.75).

ICIs did not significantly affect the incidence of any grade of radiation pneumonitis/pneumonia in patients undergoing concurrent (RR = 1.36, 95% CI, 0.76-2.45), induction (RR = 2.45, 95% CI 0.13-47.30), or adjuvant therapy (RR = 1.14, 95% CI 0.17-7.41). However, in patients receiving consolidation ICIs therapy after radiotherapy (RR = 1.30, 95% CI 1.09-1.55), there was an increased occurrence of any grade radiation pneumonitis/pneumonia. For severe radiation pneumonitis/pneumonia (Grade ≥3), neither concurrent (RR = 1.30, 95% CI 0.70-2.40), consolidation (RR = 1.02, 95% CI 0.61-1.70), induction (RR = 0.24, 95% CI 0.02-2.39), nor adjust ICIs therapy (RR = 0.50, 95% CI 0.05-5.41), showed a statistically significant impact.

ICIs treatment had no statistically significant impact on the incidence of any grade of radiation pneumonitis/pneumonia in patients with Stage I lung cancer (RR = 1.52, 95% CI 0.35-6.48) or SCLC (RR=1.50, 95% CI 0.84-2.66). However, in Stage III lung cancer patients, the addition of immunotherapy promoted the occurrence of any grade of radiation pneumonitis/pneumonia (RR = 1.21, 95% CI 1.04-1.40). For severe radiation pneumonitis/pneumonia (Grade 3 or higher), the addition of ICIs had no statistically significant impact in either in Stage III (RR = 0.97, 95% CI 0.61-1.53) or SCLC patients (RR = 1.41, 95% CI 0.79-2.52).

In our analysis, ICIs targeting different types of targets did not significantly increase the risk of any grade of radiation pneumonitis/pneumonia. Specifically, the RR were 1.26 (95% CI 0.97-1.63) for PD-L1-targeting treatments and 2.28 (95% CI 0.94-5.54) for PD-1-targeting treatments. For severe radiation pneumonitis/pneumonia (grade 3 or higher), neither PD-1-targeting treatments (RR = 0.93, 95% CI 0.10-8.31) nor PD-L1-targeting treatments (RR = 1.15, 95% CI 0.77-1.70) demonstrated a statistically significant impact on incidence (**Table 3**).

**Table 3:** Subgroup analyses of radiation pneumonia (any grade and ≥n grade) according to study characteristics and methods.

Characteristic	Radiation pneumonia (any grade)		Radiation pneumonia (≥3 grade)	
	Relative Risk	95% CI	Relative Risk	95% CI
Radiotherapy rodalities				
SBRT	1.52	[0.35, 6.48]	/	/
Con-RT	1.26	[0.96, 1.66]	1.21	[0.82, 1.77]
PRT	1.00	[0.34, 2.91]	0.42	[0.07, 2.37]
Hypo-RT	2.00	[1.13, 3,53]	1.50	[0.26, 8.75]
Timing of combination therapy with Immunotherapy				
Concurrent	1.36	[0.76,2.45]	1.30	[0.70, 2.40]
Consolidation	1.30	[1.09, 1.55]	1.02	[0.61, 1.70]
Induction	2.45	[0.13, 47.30]	0.24	[0.02, 2.39]
Adjust	0.81	[0.19, 3.43]	0.50	[0.05, 5.41]
Clinical Stage				
I	1.52	[0.35, 6.48]	/	/
III	1.21	[1.04, 1.40]	0.97	[0.61, 1.53]
Limited	1.50	[0.84, 2.66]	1.41	[0.79, 2.52]
Histology				
NSCLC	1.21	[1.04, 1.40]	0.97	[0.61, 1.53]
SCLC	1.50	[0.84, 2.66]	1.41	[0.79, 2.52]
ICI type				
PD-1	2.28	[0.94, 5.54]	0.93	[0.10, 8.31]
PD-L1	1.26	[0.97, 1.63]	1.15	[0.77, 1.70]
Total	**1.27**	**[1.12, 1.44]**	**1.12**	**[0.78, 1.60]**

SBRT, stereotactic body radiation therapy. Con-RT, conventional radiotherapy. IMRT, intensity-modulated radiation therapy. PRT, proton radiation therapy. Hypo-RT, high dose fractionated radiotherapy. SCLC, small cell lung cancer.

### 3.5 The impact of combining radiotherapy with immunotherapy on the incidence of other common adverse reactions

In the studies reviewed, our meta-analysis encompassed 18 frequent adverse reactions such as esophagitis, nausea, vomiting, decreased appetite, diarrhea, among others. Our results show that combining radiotherapy with ICIs did not statistically impact the incidence of esophagitis (RR = 0.82, 95% CI 0.20-1.56), nausea (RR = 1.06, 95% CI 0.81-1.39), decreased appetite (RR = 0.78, 95% CI 0.47-1.29), diarrhea (RR = 1.06, 95% CI 0.82-1.39), cough (RR = 1.22, 95% CI 0.79-1.86), dyspnea (RR = 1.06, 95% CI 0.84-1.34), hypoxia (RR = 1.01, 95% CI 0.80-1.27), fatigue (RR = 1.12, 95% CI 0.90-1.38), myalgia (RR = 0.93, 95% CI 0.58-1.46), arthralgia (RR = 0.59, 95% CI 0.29-1.22), back pain (RR = 0.94, 95% CI 0.62-1.42), and anemia (RR = 0.99, 95% CI 0.78-1.26). Nonetheless, the addition of ICIs treatment is associated with increases risks of fever (RR = 1.59, 95% CI 1.05-2.43), hypothyroidism (RR = 3.28, 95% CI 2.34-4.58), hyperthyroidism (RR = 3.87, 95% CI 2.27-6.62), rash (RR = 2.23, 95% CI 1.60-3.11), pruritus (RR = 2.46, 95% CI 1.68-3.60), and increased serum alanine aminotransferase (RR = 2.20, 95% CI 1.35-3.57). Furthermore, our findings also indicate that the combination of radiotherapy and ICIs does not exacerbate severe (Grade ≥3) adverse effects (**Table 4**).

**Table 4:** Adverse events experienced by lung cancer patients following treatment with ICIs and RT. .

AEs	Any Grade		≥ Grade 3	
	Relative Risk	95% CI	Relative Risk	95% CI
Esophagitis	0.82	[0.29, 1.56]	0.85	[0.17, 4.26]
Nausea	1.06	[0.81, 1.39]	0.82	[0.22, 3.03]
Decreased appetite	0.78	[0.47, 1.29]	0.51	[0.23, 1.14]
Diarrhea	1.06	[0.82, 1.39]	1.41	[0.19, 10.21]
Cough	1.22	[0.79, 1.86]	0.52	[0.21, 1.32]
Dyspnea	1.06	[0.84, 1.34]	0.97	[0.56, 1.70]
Fever	1.59	[1.05, 2.43]	1.48	[0.06, 36.18]
Fatigue	1.12	[0.90, 1.38]	0.56	[0.30, 1.05]
Myalgia	0.87	[0.55, 1.39]	1.48	[0.15, 14.13]
Arthralgia	0.67	[0.29, 1.51]	0.21	[0.02, 1.80]
Back pain	0.94	[0.62, 1.42]	0.49	[0.03, 7.84]
Hypothyroidism	3.28	[2.34, 4.58]	1.98	[0.22, 17.64]
Hyperthyroidism	3.87	[2.27, 6.62]	3.41	[0.14, 82.20]
Rash	2.23	[1.60, 3.11]	2.50	[0.53, 11.87]
Pruritus	2.46	[1.68, 3.60]	1.49	[0.06, 36.20]
Anemia	0.99	[0.78, 1.26]	0.73	[0.37, 1.42]
Serum ALT increase	2.20	[1.35, 3.57]	5.67	[0.29, 111.90]

SBRT, stereotactic body radiation therapy. Con-RT, conventional radiotherapy. IMRT, intensity-modulated radiation therapy. PRT, proton radiation therapy. Hypo-RT, high dose fractionated radiotherapy. SCLC, small cell lung cancer.

In our meta-analysis, we observed variability in the grading standards for pneumonia across the included studies, with 5 studies using CTCAE v4.0 and 9 studies using CTCAE v5.0. Stratified analyses revealed no significant differences in the risk of gradere pneumonia between these two groups (RR = 0.88, 95% CI 0.46-1.67 for CTCAE v4.0; RR = 1.25, 95% CI 0.81-1.92 for CTCAE v5.0). These findings underscore the importance of adopting a uniform grading system to reduce heterogeneity. We recommend using CTCAE v5.0 as the standard for future clinical trials to ensure consistency and comparability of results.

### 3.6 Sensitivity analysis and risk of bias

In certain studies where pneumonia incidence events are not reported, there exists a potential risk of bias affecting the analytical outcomes. To address this issue, we conducted analyses excluding studies with zero-event cases to further examine pneumonia incidence. Findings indicate that, in studies without zero-event outcomes, the inclusion of immunotherapy increases the risk of developing radiation-induced pneumonia/pneumonitis at any grade. However, there is no significant association with the risk of developing grade 3 or higher radiation-induced pneumonia/pneumonitis, which corroborates our earlier analysis (**Supplementary Figure S1**). This suggests that the presence of zero events has minimal impact on the overall effect.

To evaluate the influence of individual studies on the overall risk assessment, we performed sensitivity analyses by methodically excluding each study (**Supplementary Figures S2** and **S3**). The findings indicate that the conclusion of this meta-analysis remain stable and are not significantly impacted by specific studies.

In this meta-analysis, we employed funnel plots, Egger’s test, and Begg’s test to assess publication bias. The results revealed no significant discrepancies between the outcomes of this meta-analysis and the initial findings, affirming the robustness and reliability of our results (**Supplementary Figure S4**).

## 4 Discussion

This study presents a meta-analysis of 15 studies involving 3,769 patients to evaluate the efficacy of radiotherapy or chemoradiotherapy alone versus in combination with ICIs in the treatment of lung cancer patients, without significantly increasing the risk of severe (grade ≥3) pneumonitis. In the management of locally advanced NSCLC, chemoradiotherapy has proven substantially more effective than chemoradiotherapy or radiotherapy alone. PACIFIC and GEMSTONE-301 have established new standards for advanced NSCLC treatment by notably improving survival outcomes. Our analysis corroborates that combining radiotherapy with ICIs significantly improved PFS and OS. This survival benefit is underpinned by a synergistic interplay between radiotherapy and ICIs. Radiotherapy acts as an *in situ* vaccine, inducing immunogenic cell death and activating the cGAS-STING pathway to stimulate type I interferon (IFN-α/β) production, which enhances dendritic cell maturation and tumor antigen presentation (6). Furthermore, radiotherapy promotes epitope spreading and increases the diversity of tumor-specific T-cell clones, thereby broadening the immune response (7). Concurrently, the resulting upregulation of PD-L1 in the tumor microenvironment provides a critical target for ICIs to reverse T-cell exhaustion and unleash a robust, systemic anti-tumor immune response.

Subgroup analyses revealed that the survival benefit of combining radiotherapy with ICIs is modulated by both technical and biological factors. Regarding radiotherapy technique, SBRT and hypo-fractionated radiotherapy regimens were associated with improved PFS, likely due to their high dose per fraction inducing more potent immunogenic cell death. Conventional fractionation improved both PFS and OS, possibly owing to its broader application in curative-intent settings for locally advanced disease. In contrast, PRT combined with ICIs did not confer a significant survival benefit. The proposed “sparing effect” of PRT, while protecting normal tissues, may also preserve immunosuppressive cells like Tregs within the tumor microenvironment (8). Furthermore, the sharp dose fall-off might lead to insufficient immunogenic cell death at the expanding tumor periphery, failing to initiate a robust systemic immune response.

The timing of ICI administration relative to radiotherapy emerged as a critical determinant of efficacy. Consolidation immunotherapy following radiotherapy consistently improved OS. For instance, the PACIFIC-2 study did not demonstrate substantial improvement by adding durvalumab to standard concurrent chemoradiotherapy. Although patients with EGFR mutations were reported, 143 participants had unknown genetic statuses, potentially influencing study outcomes (9). This sequence allows radiotherapy to first remodel the tumor microenvironment—releasing tumor antigens and inducing inflammatory signalsatorym then primes a systemic immune response that can be effectively amplified by subsequent ICIs (10). Conversely, concurrent administration showed no significant advantage over radiotherapy alone. A plausible explanation is that the radiotherapy itself may deactivate the very effector T cells that are recruited and activated by concurrent ICIs, a phenomenon of “in-field lymphodepletion” (11). Moreover, induction immunotherapy prior to radiotherapy yielded predominantly negative results. Administering ICIs in a non-inflamed, immunosuppressive tumor microenvironment can precipitate T-cell exhaustion in the absence of a strong antigenic stimulus. Perhaps more critically, this sequence may select for pre-existing immune-resistant tumor clones, which then repopulate the tumor following radiotherapy, ultimately leading to treatment failure (12).

The impact of different radiotherapy techniques on the incidence of radiation pneumonitis/pneumonia is a clinically significant concern. Our analysis revealed that the combination of radiotherapy with ICIs did not significantly increase the incidence of grade ≥radeencently compared to radiotherapy alone. This conclusion holds across most radiotherapy techniques analyzed, including SBRT, conventional and PRT radiotherapy. First, advancements in radiotherapy technique have fundamentally reduced the baseline risk of severe lung toxicity. The physical precision of modalities like PRT, which leverages the Bragg peak to minimize exit dose to normal lung (13, 14), and SBRT, characterized by its high conformity and rapid dose fall-off, ensures that a smaller volume of healthy lung tissue is exposed to significant radiation doses (15). Second, complex immunomodulatory interactions, rather than simple toxicity addition, likely occur. Radiotherapy may systemically induce immunosuppressive regulatory T cells (Tregs) or myeloid-derived suppressor cells (MDSCs) via an “abscopal-like” effect, which could paradoxically protect normal tissues from ICI-driven inflammation (16). Alternatively, localized radiation may deplete the very pool of activated T cells within the irradiation field that could otherwise migrate and attack normal lung parenchyma. Furthermore, the release of a broad repertoire of tumor neoantigens by radiotherapy may effectively “focus” the immune response away from autoimmunity and toward the tumor itself. Nevertheless, so far, only the study by liu et al. has reported the hypo-fractionated radiotherapy is associated with an increased risk of radiation pneumonitis/pneumonia in lung cancer patients undergoing treatment with ICIs (17). The results of this study require further validation with additional clinical data.

Emerging data from RCTs on SCLC patients receiving radiotherapy combined with ICIs indicate that adding immunotherapy does not improve PFS or OS in extensive-stage SCLC (ES-SCLC) patients. Our findings reveal that the addition of immunotherapy does not improve OS in SCLC patients receiving radiotherapy or chemoradiotherapy. However, it may potentially have a clinical impact on progression-free survival in these patients. Nonetheless, this treatment approach does not significantly affect radiation pneumonitis/pneumonia incidence at any severity level. The NRG LU005 study showed no significant improvement in survival (18). Due to limitations in the number of RCTs on SCLC, further validation with larger samples is essential. We anticipate comprehensive disclosure of Phase III clinical data from studies like DeLLphi-306, HEYLYNK-013, and HLX10-020.

Recent years have positioned the PD-1/PD-L1 axis as a significant clinical target for cancer therapy. Our meta-analysis found that both PD-1 and PD-L1 inhibitors effectively improved PFS, with PD-L1 inhibitors also benefiting OS. Immature or undisclosed OS data in the PD-1 subgroup could explain this disparity. Previous meta-analyses on NSCLC with PD-1 and PD-L1 inhibitors (19) demonstrated statistically significant associations between PD-1 inhibitors and increased pneumonia incidence at any level, including grades 3 and 4 (20, 21). However, our study observed that PD-1 and PD-L1 monoclonal antibodies did not significantly increase pneumonia incidence, warranting cautious interpretation due to relatively small sample sizes.

Our meta-analysis revealed no significant differences in the risk of grade ≥r pneumonia between studies using CTCAE v4.0 and those using CTCAE v5.0. This finding underscores the importance of adopting a uniform grading system to reduce heterogeneity and ensure consistency in reporting adverse events. We recommend using CTCAE v5.0 as the standard for future clinical trials to enhance comparability of results. This approach will facilitate more reliable meta-analyses and improve the generalizability of findings across different studies.

To our knowledge, this study represents the most current and comprehensive meta-analysis of RCTs, providing robust evidence on the efficacy of radiotherapy or chemoradiotherapy combined with ICIs. Our focuses were particularly on the risk of developing radiation pneumonitis or pneumonia following such treatments. This analysis encompasses data from numerous large-scale clinical studies and RCTs concerning SCLC, expanding the scope of our findings compared to prior meta-analyses. Furthermore, in this study, some reports indicated zero pneumonia events, which could potentially affect the analysis outcomes. By conducting additional analyses that exclude studies with zero events, along with sensitivity analyses, we conclude that the impact of zero events on the overall results of this study is minimal. The results demonstrate a degree of robustness.

This study also has some limitations. First, although we endeavored to select accurate data on radiation pneumonitis by examining reported treatment-related adverse events (TRAEs), not all studies explicitly differentiated between radiation pneumonitis and pneumonia. Specifically, five studies did not provide distinct data for radiation pneumonitis, necessitating the use of pneumonia data as a proxy. This substitution may have introduced bias into our findings on radiation pneumonitis. Second, one notable limitation is the lack of detailed baseline data distinguishing induction and consolidation period patients. The differences in baseline characteristics, such as tumor stage, PD-L1 expression, and prior treatment history, may influence treatment responses. Additionally, small sample sizes in some subgroups, particularly in PRT and induction immunotherapy, may have led to insufficient statistical power, limiting our ability to detect significant effects. Future studies should address these limitations by providing detailed baseline data and increasing sample sizes, especially in subgroups with inconclusive results. Third, evidence for SCLC remains limited. All included studies focused on limited-stage SCLC, and the lack of significant benefits observed may be attributable to insufficient sample size and the absence of data on extensive-stage SCLC. Furthermore, we attempted to explore heterogeneity through subgroup analyses but did not fully address all potential confounding factors, such as detailed patient baseline characteristics (e.g., tumor mutational burden, PD-L1 expression). These factors could influence treatment responses and contribute to heterogeneity. Future studies should include comprehensive data on these variables to enhance the robustness of heterogeneity analyses and identify subgroups that may benefit most or be at higher risk.

We acknowledge the heterogeneity in immune effects across different disease stages and radiotherapy regimens. To advance the field, future research must transition to a precision medicine approach. This entails the development of predictive biomarkers, such as dynamic changes in circulating cytokines (e.g., T-cell recruiting CXCL9/10 versus pro-inflammatory IL-6/IL-8) and the peripheral T-cell repertoire diversity, to stratify patients for benefit and pneumonitis risk (22, 23). Furthermore, resolving the optimal sequencing of radiotherapy and ICIs is a critical unknown (24). Preclinical evidence suggests timing profoundly impacts dendritic cell cross-priming and PD-L1 induction (25); this must be tested in prospectively designed clinical trials with integrated translational components. Integrating detailed radiotherapy parameters with such immunological data will be key to tailoring combination therapy.

## 5 Conclusion

In summary, this study indicates that compared to radiotherapy or chemoradiotherapy alone, PD-1/PD-L1 inhibitors significantly improve the PFS and OS of lung cancer patients. Although the addition of PD-1/PD-L1 inhibitors can to some extent increase the risk of developing pneumonia at all grades, they do not affect the incidence of severe radiation pneumonitis/pneumonia (Grade 3 or higher), and their safety remains within clinically controllable limits.

## Data Availability

Publicly available datasets were analyzed in this study. All data relevant to the study are included in the article or uploaded as **Supplementary Information**.
