# Supplementary material for: Enhanced efficacy of lung cancer treatment with radiotherapy and immune checkpoint inhibitors without increased pneumonia risk: a systematic review and meta-analysis of randomized controlled trials
Source: Front Immunol. 2025 Dec 8;16:1685963. doi: 10.3389/fimmu.2025.1685963 (PMC12719472; doi:10.3389/fimmu.2025.1685963)
Supplement: Supplementary file 1 [file DataSheet1.docx]

**Supplementary File**

**
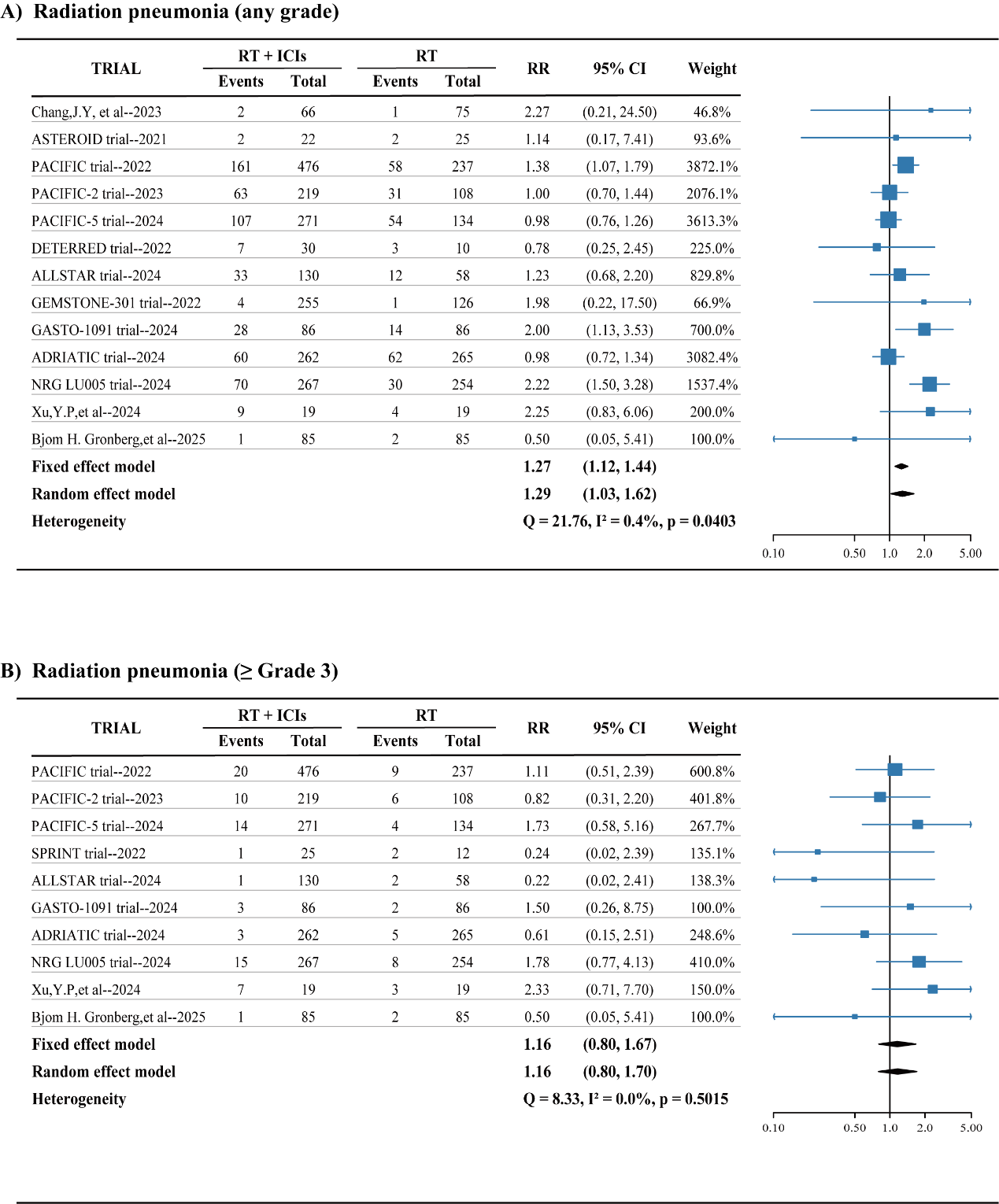
**

**FIGURE S1 Pooled Risk Ratios (RRs) of radiation pneumonia Across Randomized Clinical Trials (deleted studies that contain zero events).**

**
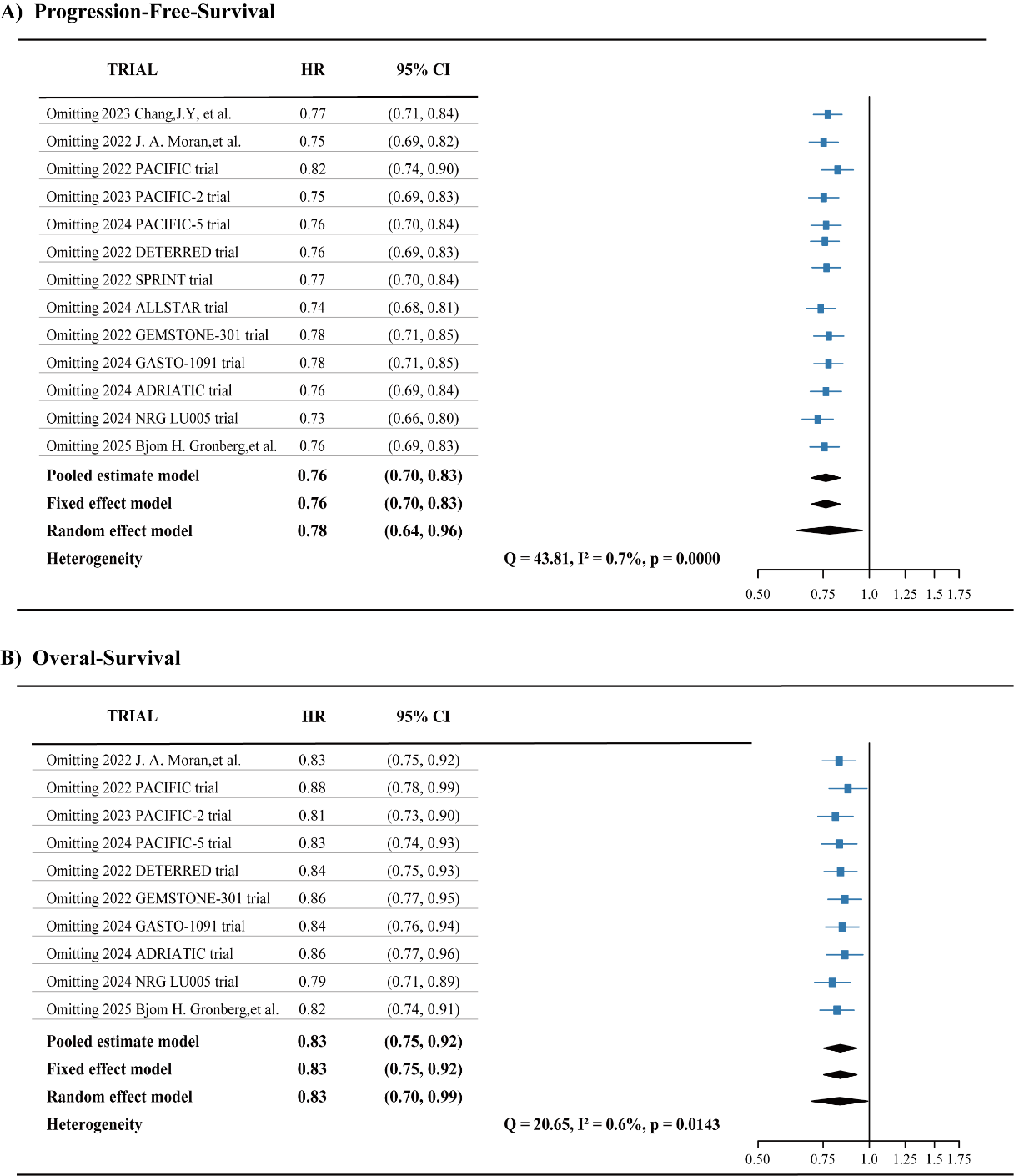
**

**FIGURE S2 Sensitivity Analysis of** **Progression-Free-Survival and** **Overall-Survival Across Randomized Clinical Trials.**

**
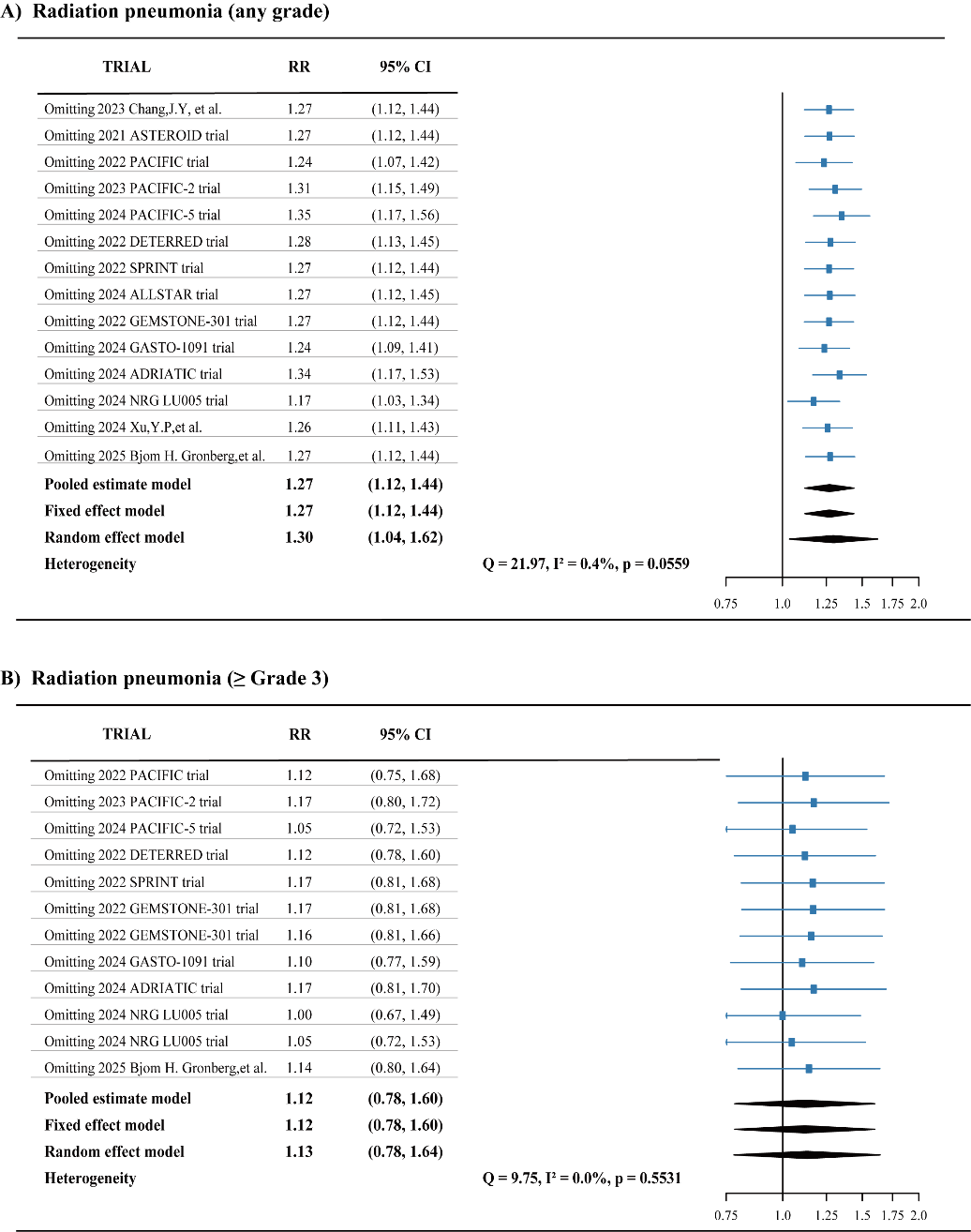
**

**FIGURE S3 Sensitivity Analysis of Radiation Pneumonia Across Randomized Clinical Trials.**

**
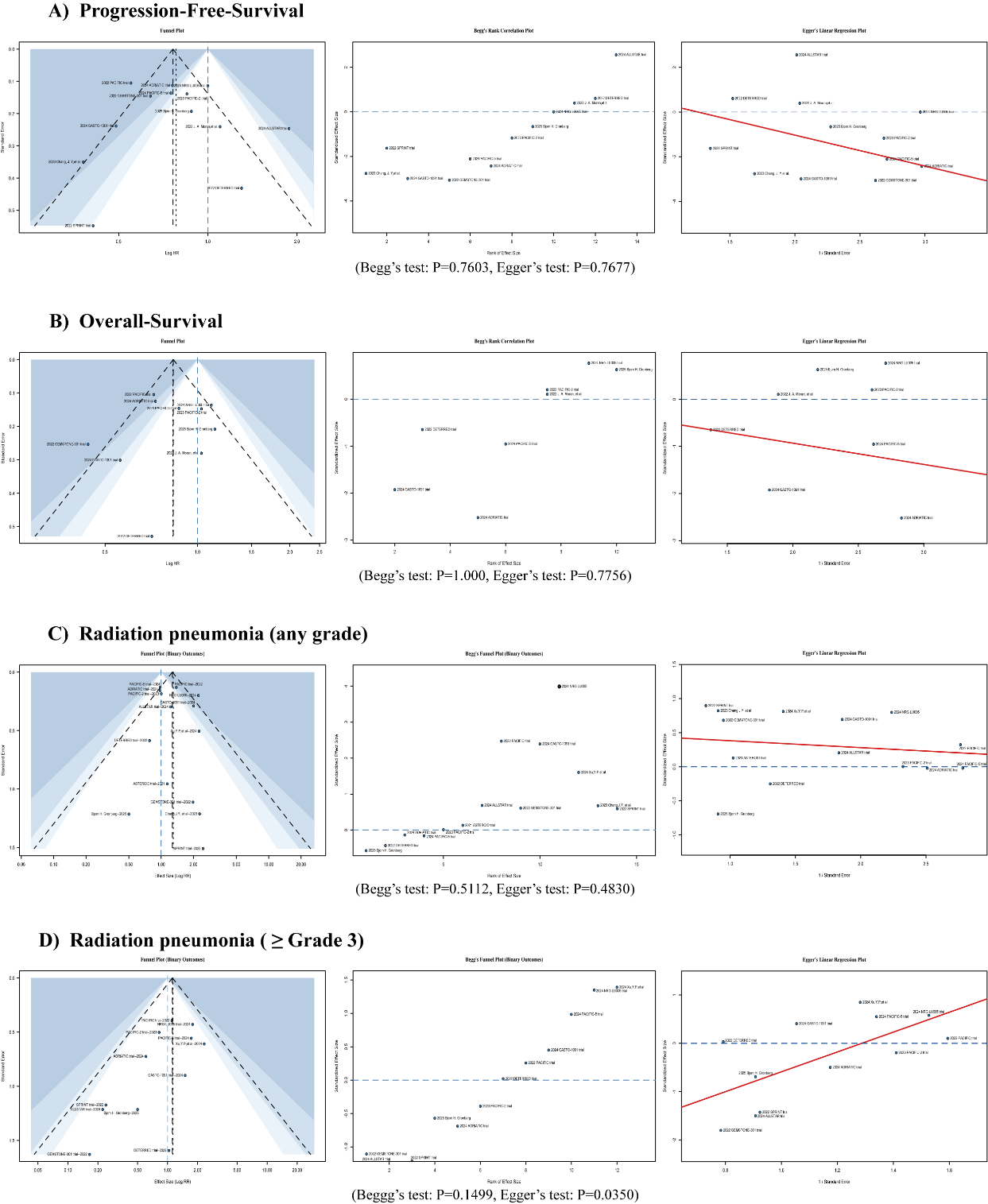
**

**FIGURE S4 Risk of Bias of Survival Outcomes and Radiation Pneumonia Across Randomized Clinical Trials.**

**
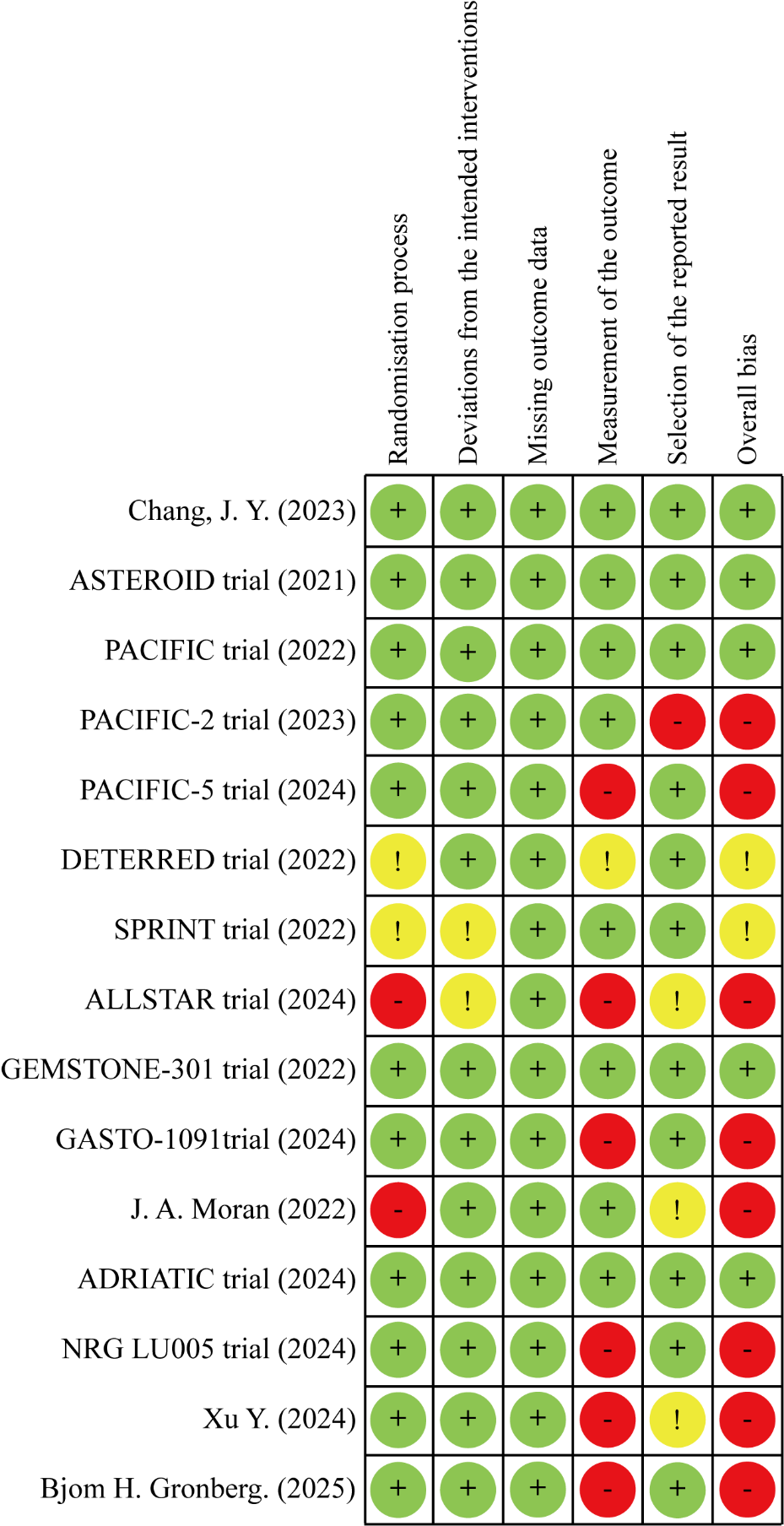
**

**FIGURE S5 Risk of bias summary.**
